# Differences in Knowledge, Awareness, Practice, and Health Symptoms in Farmers Who Applied Organophosphates and Pyrethroids on Farms

**DOI:** 10.3389/fpubh.2022.802810

**Published:** 2022-02-02

**Authors:** Ajchamon Thammachai, Ratana Sapbamrer, Juthasiri Rohitrattana, Siam Tongprasert, Surat Hongsibsong, Kampanat Wangsan

**Affiliations:** ^1^Department of Community Medicine, Faculty of Medicine, Chiang Mai University, Chiang Mai, Thailand; ^2^Center for Safety, Health and Environment of Chulalongkorn University, Bangkok, Thailand; ^3^Department of Rehabilitation Medicine, Faculty of Medicine, Chiang Mai University, Chiang Mai, Thailand; ^4^School of Health Sciences Research, Research Institute for Health Sciences, Chiang Mai University, Chiang Mai, Thailand

**Keywords:** practice, health symptoms, organophosphates, pyrethroids, pesticides, farmers, knowledge, awareness

## Abstract

**Objective:**

This cross-sectional study aimed to examine farmers' knowledge, awareness, practices regarding pesticide use, and prevalence of health symptoms related to pesticides exposure among farmers who applied organophosphates (OP) and pyrethroids (PY).

**Methods:**

Data regarding demographic variables and health symptoms pertinent to pesticide use was collected from 67 farmers who applied OP and 50 farmers who applied PY using interviews from January to March 2021.

**Results:**

The farmers who applied OP had lower knowledge, awareness, and prevention practices regarding pesticide use than those who applied PY. After adjustment of covariate variables, the farmers who applied OP had a significantly higher prevalence of respiratory conditions (OR = 8.29 for chest pain, OR = 6.98 for chest tightness, OR = 27.54 for dry throat, and OR = 5.91 for cough), neurological symptoms (OR = 10.62 for fatigue and OR = 6.76 for paresthesia), and neurobehavioral symptoms (OR = 13.84 for poor concentration, OR = 3.75 for short term memory, and OR = 8.99 for insomnia) related to pesticide exposure than those who applied PY.

**Conclusion:**

Our findings suggest that OP had a more adverse effect on human health than PY, resulting in a higher prevalence of pesticide-related symptoms. The outcomes of this study have the benefit of providing vital information for all stakeholders with regard to the implementation of safe practices in the utilization of personal protective equipment (PPE) and pesticide use in a health intervention and health promotion program.

## Introduction

Thailand's government has made efforts to enhance cro*p* yields in recent decades (1). As a result, the importing of pesticides has steadily increased year on year to facilitate control of pests, including insects, weeds, and fungi (2, 3). The most commonly imported insecticide classes were organophosphates (OP) such as chlorpyrifos (1,723.02 tons) and pirimiphos-methyl (750.46 tons), followed by pyrethroids (PY) such as cypermethrin (675.52 tons) (1). The toxicity of both OP and PY can pollute the environment and pose a risk to animal and human health (4). Pesticides exposure may occur ingested, absorbed through the skin, or inhaled (5, 6). Most health concerns arise from farmers' exposure to pesticides via cutaneous and inhalation pathways when applying pesticides, whereas consumer ingestion of pesticide-contaminated food (7). Farmers may pose a higher health risk than consumers since they are routinely exposed to higher doses of pesticides (5, 8).

Pesticide exposure can have both acute and chronic health effects (5). The acute effects include blurred vision, vomiting, nausea, dizziness, cramp, numbness, and muscle weakness (8, 9). As regards chronic health effects, the evidence is inconsistent, and the mechanisms involved are unclear (7, 8). In several human studies, pesticides have been shown to be related to chronic adverse effects on the immune, neurological, respiratory, endocrine, and reproductive systems (7, 10–16). Symptoms indicating neurobehavioral, motor, and sensory dysfunction, have been reviewed in some symptom prevalence studies (11, 17–19).

Several studies indicate that OP has a negative impact on human health, nevertheless, the data pertinent to PY toxicity is inconsistent (20, 21). Organophosphate inhibits acetylcholinesterase leading to excessive activity at acetylcholine receptors. Pyrethroids act on sodium channels by delaying voltage-sensitive sodium channel closure (20). Even though PY are generally less hazardous than OP, poisoning with a high amount can be fatal (22). Some studies have shown that PY toxicity might have symptoms that are similar to organophosphate poisoning (20). Therefore, the impact of PY on human health is raising concern. Most previous studies on Thai farmers have been limited to reports of single pesticide exposure despite many farmers using a range of pesticides in the cultivation of multiple crops (2). To date, no studies comparing pesticide-related health problems among Thai farmers exposed to both OP and PY have been reported. Only one study has evaluated the effect of childhood exposure to OP and PY on neurobehavior, but results were not significant predictors of adverse neurobehavioral performance (23). They suggest that the impact of these pesticides might be dependent on the half-life of OP and PY (23). Therefore, the use of a questionnaire to assess the relationshi*p* between chronic health effects and pesticide exposure is warranted (23).

Previous studies provide evidence that perception influences the use of personal protective equipment (PPE) and pesticide safety practices (24). In reality, improper PPE and unsafe practices are frequently reported, information from some studies indicating factors affecting the use of PPE and pesticide safety practices are inadequate and inconsistent (24). Hence, understanding the reasons behind the knowledge, awareness, and practices regarding pesticide use is vital in order to facilitate the design of interventions to minimize exposure of pesticides among farmers.

To understand the extent of problem the prevalence of use of both OP and PY pesticides among Thai farmers is important. Therefore, the purpose of this study was to compare the knowledge, awareness, practices, and health symptoms associated with pesticide exposure in farmers who applied OP and PY in farms. The result of this study is useful to provide information for health surveillance systems and health intervention to raise safety awareness among farmers in the future.

## Materials and Methods

### Participants

**Figure 1:**
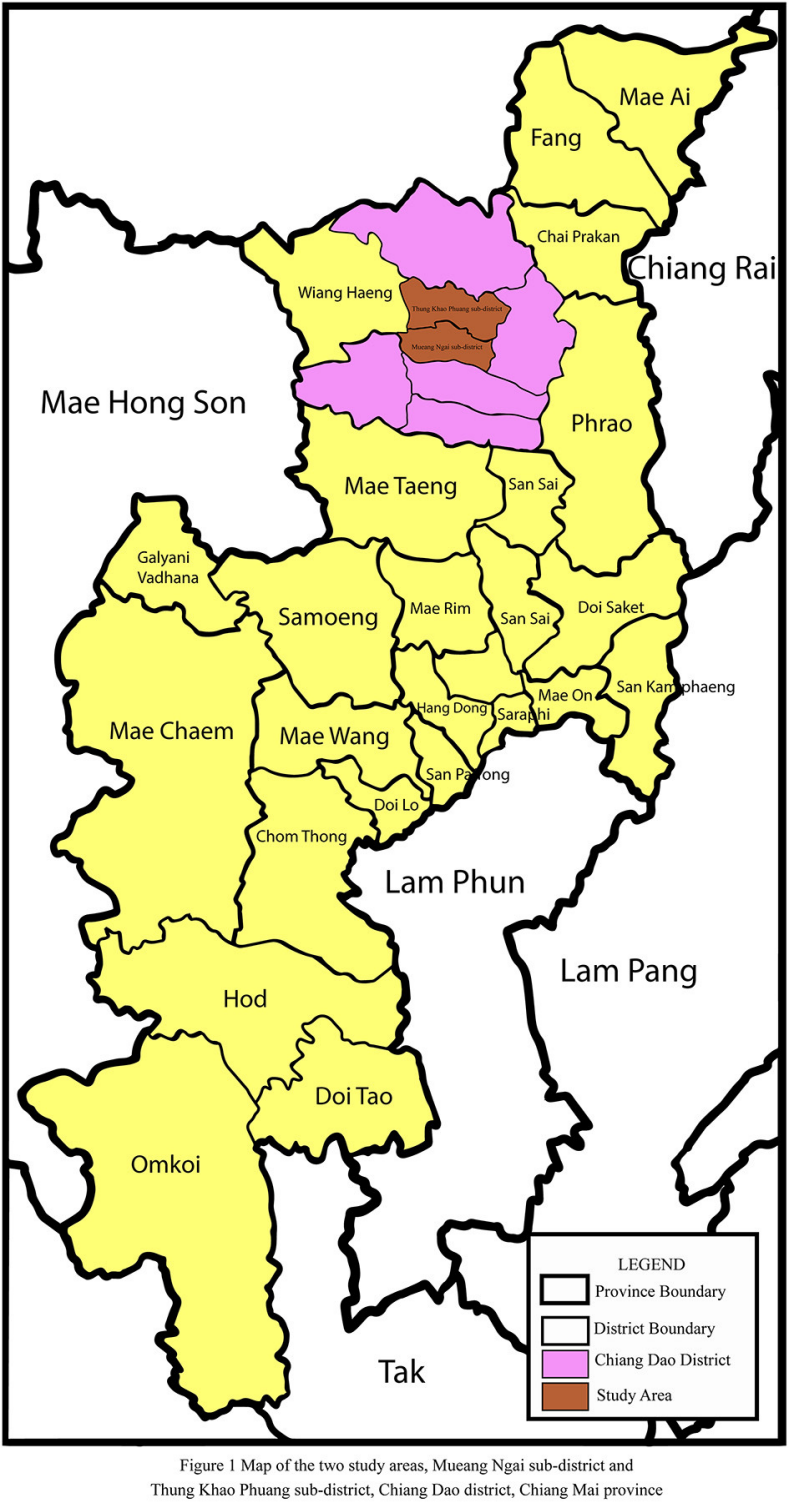
Ma*p* of the two study areas, Mueang Ngai sub-district and Thung Khao Phuang sub-district, Chiang Dao district, Chiang Mai province.

This was a population-based cross-sectional study of farmers who were actively participating in farming production. Two subdistricts of the Chiang Dao district were chosen at random. Mueang Ngai and Thung Khao Phuang sub-districts, both in Chiang Dao district, Chiang Mai province, are shown in Figure 1. The socio-cultural contexts of all of these subdistricts were identical. The number of participants was decided by the number of pesticide-related hospitalizations in each subdistrict's health-promoting hospitals. Participants included farmers who used a single pesticide, such as OP or PY pesticides. The participants were 18 to 70 years old and had lived in the Mueang Ngai sub-district, or Thung Khao Phuang sub-district, Chiang Dao district, Chiang Mai province, northern Thailand, for at least 1 year. Farmers with underlying diseases including respiratory and cardiovascular disease, cancer, diabetes, or neurological problems were excluded. Those who met the inclusion criteria (*n* = 138) included 83 farmers who applied OP (67 males, 16 females) and 55 farmers who applied PY (28 males, 27 females). Of these 138 individuals, 67 farmers who applied OP [51 (76.1%) males, 16 (23.9%) females] and 50 farmers who applied PY [27 (54.0%) males, 23 (46.0%) females] agreed to volunteer as study subjects and signed a written consent form. Data were collected through interview forms from January to March 2021, which is the period when pesticides are applied on farms for pest control.

### Interviews

The principal investigator trained all staff members before the participants were interviewed. The validated interview form was a structured questionnaire which was developed based on the manual for the Occupational Health Service Agricultural Health Clinics (Bureau of Occupational and Environmental Diseases, Center for Disease Control, 2015) and Department of Disease Control, Ministry of Public Health (MOPH) Thailand and from similar published studies (7, 11). Three experts in environmental and occupational health assessed the questionnaire's content validity, language suitability, and scoring criteria. The item-objective congruence index (IOC) was used to assess the content validity of questionnaires. The average IOC score of knowledge, awareness, practice regarding pesticide use, and health symptom related to pesticide exposure were 0.89, 0.93, 0.98, and 0.97 respectively. These results show that the questions are consistent with the objectives.

The questionnaire had five sections. The first section consisted of demographic details including age, education status, nationality, income, smoking status, alcohol drinking, and body mass index (BMI). The second section had five questions related to agricultural information including the number of years of farm work, frequency of pesticide use, working hours, type of sprayer, and the distance between farm and residence. The third section had 17 questions to assess knowledge and awareness regarding pesticide use. The fourth section included questions regarding practice, such as the use of PPE during pesticide application and pesticide exposure prevention. The responses were recorded as “yes” or “no” responses. A score of “1” was provided for each correct response, while a score of “0” was given for each incorrect response. In the fifth section, questions regarding health symptoms within 1 month of enrollment were asked. The questions were presented as 30 items in six sections focusing on: (1) respiratory tract symptoms (difficulty in breathing, chest pain, chest tightness, heart palpitation, dry throat, cough); (2) musculoskeletal symptoms (numbness, cramp, muscle weakness); (3) neurological symptoms (headache, dizziness, vomiting, fatigue, eye twitches, hand tremors, dysesthesia, paresthesia); (4) epithelial/mucosal surface symptoms (eye irritation, ulcer/blister, itchy, sweating); (5) neurobehavioral symptoms (poor concentration, short term memory, compulsion, depression, insomnia); (6) other symptoms (blurry vision, diarrhea, stomach ache, decreased sex drive).

### Statistical Analysis

Data pertinent to demographic characteristics, knowledge, awareness, and practices regarding pesticide use were analyzed by using frequency, mean, median, standard deviation, range, and percentile (P25th-P75th). All parameters were tested for normality using the Kolmogorov-Smirnov and Shapiro-Wilk tests. Comparison of demographic characteristics, agricultural information between farmers who applied OP and PY were performed using Chi-squared and independent sample *t*-tests. Due to the non-normal distribution of variables, the Mann-Whitney *U* test was used to compare agricultural information between farmers who applied OP and PY. Comparison of knowledge, awareness, and practices regarding pesticide use between farmers who applied OP and PY were performed using Chi-square. The odds ratios (OR) and 95% confidence intervals were calculated to assess symptoms related to pesticide exposure. The results were obtained from univariate analysis and multivariate logistic regression in a model adjusted for age, gender, nationality, body mass index, smoking status, alcohol consumption, pesticide group, frequency of pesticide use, the number of years of farm work, knowledge, awareness, and practice scores. *p-value*s of < 0.05 were accepted as statistically significant.

## Results

Table 1 shows the demographic characteristics among farmers who applied OP and PY. The majority of the farmers were male with a mean age of 48.3 ± 12.5 years. There were no statistical differences between the two groups in terms of education, nationality, income, smoking status, age, and BMI. However, there was a significantly higher number of men in the farmers who applied OP and a significantly higher level of alcohol use than in those who applied PY.

**Table 1:** Demographic characteristics among farmers who applied OP and PY (*n* = 117).

**Parameters**		**Total (*n =* 117)**	**Farmers who applied OP (*n =* 67)**	**Farmers who applied PY (*n =* 50)**	***p-value***
Gender, *n* (%)^a^	Male	78 (66.7)	51 (76.1)	27 (54.0)	0.021^*^
	Female	39 (33.3)	16 (23.9)	23 (46.0)	
Education, *n* (%)^a^	No education	25 (21.4)	18 (26.9)	7 (14.0)	0.260
	Primary level	51 (43.6)	27 (40.3)	24 (48.0)	
	Secondary level	35 (29.9)	20 (29.9)	15 (30.0)	
	Bachelor level	6 (5.1)	2 (3.0)	4 (8.0)	
Nationality, *n* (%)^a^	Thai	85 (72.6)	44 (65.7)	41 (82.0)	0.080
	Other	32 (27.4)	23 (34.3)	9 (18.0)	
Income (baht), *n* (%)^a^	<4,500	38 (32.5)	19 (28.4)	19 (38.0)	0.391
	4,500–10,000	44 (37.6)	26 (38.8)	18 (36.0)	
	10,000–15,000	26 (22.2)	18 (26.9)	8 (16.0)	
	> 15,000	9 (7.7)	4 (6.0)	5 (10.0)	
Smoking status, *n* (%)^a^	Yes	37 (31.6)	23 (34.3)	14 (28.0)	0.598
	No	80 (68.4)	44 (65.7)	36 (72.0)	
Alcohol drinking, *n* (%)^a^	Yes	51 (43.6)	40 (59.7)	11 (22.0)	<0.001^**^
	No	66 (56.4)	27 (40.3)	39 (78.0)	
Age (years)^b^	Mean ± SD	48.3 ± 12.5	47.9 ± 11.7	48.6 ± 13.5	0.789
	(Min, Max)	(20.0, 67.0)	(21.0, 67.0)	(20.0, 67.0)	
Body mass index^b^	Mean ± SD	23.5 ± 4.1	22.9 ± 3.4	24.3 ± 4.9	0.066
	(Min, Max)	(16.1, 35.7)	(17.3, 32.6)	(16.1, 35.7)	

*OP, organophosphates; PY, pyrethroids; SD, standard deviation; Min, minimum; Max, maximum; kg, kilogram; cm, centimeter;*

* *p < 0.05;*

** *p < 0.01;*

a *obtained from Chi-square;*

b *obtained from Independent sample t-test*.

Table 2 presents the agricultural information among farmers who applied OP and PY. 54.7% of the farmers applied pesticides one to two times per week and had farm work experience of 16.2 ± 10.5 years. Most farmers had working hours of 5–8 h per day (65.8%), and use a knapsack sprayer (83.8%). There were no statistical differences regarding the agricultural information between the two groups of farmers who applied OP and PY.

**Table 2:** Agricultural information of farmers who applied OP and PY (*n* = 117).

**Parameters**		**Total (*n =* 117)**	**Farmers who applied OP (*n =* 67)**	**Farmers who applied PY (*n =* 50)**	***p-*value**
Frequency of pesticide use, *n* (%) ^a^	2 times/month or less	53 (45.3)	27 (40.3)	26 (52.0)	0.285
	1-2 times/week	64 (54.7)	40 (59.7)	24 (48.0)	
Years in farm work (years) ^b^	Mean ± SD	16.2 ± 10.5	15.9 ± 10.9	16.6 ± 10.1	0.484
	Median (P^25th^, P^75th^)	15.0 (8.0, 20.0)	20.0 (8.0, 20.0)	19.0 (8.0,20.0)	
Working hours, *n* (%) ^a^	2–4 h	16 (13.7)	10 (14.9)	6 (12.0)	0.881
	5–8 h	77 (65.8)	43 (64.2)	34 (68.0)	
	>8 h	24 (20.5)	14 (6.9)	10 (20.0)	
Type of sprayer, *n* (%) ^a^	Knapsack sprayer	98 (83.8)	53 (79.1)	45 (90.0)	0.184
	Machine sprayer	19 (16.2)	14 (20.9)	5 (10.0)	
Distance between farm and residence, *n* (%) ^a^	<3 km	69 (51.0)	40 (59.7)	29 (58.0)	0.853
	>3 km	48 (41.0)	27 (40.3)	21 (42.0)	

*OP, organophosphates; PY, pyrethroids; SD, standard deviation; P^25th^, 25^th^ percentile; P^75th^, 75^th^ percentile;*

* *p < 0.05;*

** *p < 0.01;*

a *obtained from Chi-square;*

b *obtained from Mann-Whitney U test*.

The knowledge and awareness regarding pesticide use among farmers who applied OP and PY are presented in Table 3. The issues that the farmers who applied PY scored significantly higher knowledge than those who applied OP are as follows: route of entry of pesticide; mixing pesticide as described on the recommendations on the label; an adverse effect of pesticides on animal health and environment. The farmers who applied PY scored significantly higher awareness than those who applied OP are as follows: showering and changing the clothes after applying pesticides, using PPE, and not washing the spray tank in a river or waterway.

**Table 3:** Knowledge and awareness regarding pesticide use among farmers who applied OP and PY (*n* = 117).

**Statement**	**N (%) who answer correct**			
	**Total (*n =* 117)**	**Farmers who applied OP**	**Farmers who applied PY**	***p-*value**
		**(*n =* 67)**	**(*n =* 50)**	
**Knowledge**
You should select an appropriate pesticide that is specific to the insects.	112 (95.7)	62 (92.5)	50 (100.0)	0.070
Pesticides can enter into the body through ingestion, inhalation, and dermal contact.	28 (23.9)	11 (16.4)	17 (34.0)	0.047^*^
Pesticides must be mixed according to the label's recommendations.	49 (41.9)	18 (26.9)	31 (62.0)	<0.001^**^
Pesticides have an adverse effect on human health.	79 (67.5)	41 (61.2)	38 (76.0)	0.136
Pesticides have an adverse effect on animal health.	99 (84.6)	52 (77.6)	47 (94.0)	0.030^*^
Pesticides have an adverse effect on the environment.	108 (92.3)	58 (86.6)	50 (100.0)	0.019^*^
If pesticides splashed into eyes, you should wash your eyes with water immediately.	108 (92.3)	60 (89.6)	48 (96.0)	0.345
While spraying pesticides, you must wear a mask or a respirator.	114 (97.4)	64 (95.5)	50 (100.0)	0.260
Spraying pesticides at noon is more hazardous to health than spraying pesticides in the morning.	72 (61.5)	43 (64.2)	29 (58.0)	0.626
You should change your clothes after applying pesticides.	51 (43.6)	32 (47.8)	19 (38.0)	0.387
Empty pesticide containers should be disposed of by burying in the ground.	36 (30.8)	21 (31.3)	15 (30.0)	0.507
**Awareness**				
Pesticides are unnecessary for increasing productivity.	110 (94.0)	63 (94.0)	47 (94.0)	0.995
You should shower and change your clothes after applying pesticides.	91 (77.8)	44 (65.7)	47 (94.0)	0.001^**^
You should not drink or eat while applying pesticides.	111 (94.9)	64 (95.5)	47 (94.0)	0.712
It is necessary to use personal protection equipment (PPE) while applying pesticides.	59 (50.4)	17 (25.4)	42 (84.0)	<0.001^**^
It is easy and practical to wear PPE while applying pesticides.	74 (63.2)	45 (67.2)	29 (58.0)	0.410
The spray tanks should not be washed in a river or waterway.	68 (58.1)	29 (43.3)	39 (78.0)	<0.001^**^

*The results were obtained from Chi-square and presented as number (n) and percentage (%) in parenthesis; OP= organophosphates; PY = pyrethroids;*

* *p < 0.05;*

** *p < 0.01*.

Table 4 shows the practices regarding pesticide use among farmers who applied OP and PY. The issues that the farmers who applied OP had lower significantly prevention practices in than those who applied PY are as follows: buying pesticides after survey; choosing pesticides that are labeled; reading the directions on the pesticides label; mixing pesticides as recommended on the label; using gloves when mixing pesticides; and not eating food and drinking water whilst working with pesticides.

**Table 4:** Practices regarding pesticide use among farmers who applied OP and PY (*n* = 117).

**Practice statement**	**Exposure prevention practices**			***p-*value**
	**Total**	**Farmers who applied OP**	**Farmers who applied PY**	
	**(*n =* 117)**	**(*n =* 67)**	**(*n =* 50)**	
**Before application**
Survey type of pests before buying pesticides	97 (82.9)	51 (76.1)	46 (98.0)	0.045^*^
Choose pesticides that are labeled	97 (82.9)	50 (74.6)	47 (94.0)	0.012^*^
Read the directions on the pesticide label	91 (77.8)	44 (65.7)	47 (94.0)	0.001^**^
Mix pesticides as label prescription	96 (82.1)	50 (74.6)	46 (98.0)	0.029^*^
Mix pesticides outdoors	116 (99.1)	66 (98.5)	50 (100.0)	0.386
Check spraying equipment	114 (97.4)	64 (95.5)	50 (100.0)	0.355
Use gloves when mixing pesticides	86 (73.5)	43 (64.2)	43 (86.0)	0.015^*^
**During application**
Wear gloves	112 (95.7)	63 (94.0)	49 (98.0)	0.556
Wear boots	116 (99.1)	67 (100.0)	49 (98.0)	0.427
Wear long-sleeved shirt	117 (100.0)	67 (100.0)	50 (100.0	–
Wear long pants	117 (100.0)	67 (100.0)	50 (100.0)	–
Wear hat	114 (97.4)	64 (95.5)	50 (100.0)	0.260
Wear oral or nose mask	113 (96.6)	65 (97.0)	48 (96.0)	0.765
Wear goggles	12 (14.3)	10 (14.9)	2 (4.0)	0.105
Spray upwind	117 (100.0)	67 (100.0)	50 (100.0)	–
Do not eat food or drink	65 (55.6)	24 (35.8)	41 (82.0)	<0.001^**^
**After application**
Change clothes immediately	7 (6.0)	4 (6.0)	3 (6.0)	0.995
Shower immediately	13 (11.1)	10 (14.9)	3 (6.0)	0.222
Wash equipment before storing	56 (47.9)	32 (47.8)	24 (48.0)	0.980

*The results were obtained from Chi-square and presented as number (n) and percentage (%) in parenthesis; OP, organophosphates; PY, pyrethroids;*

* *p < 0.05;*

** *p < 0.01*.

**Figure 2:**
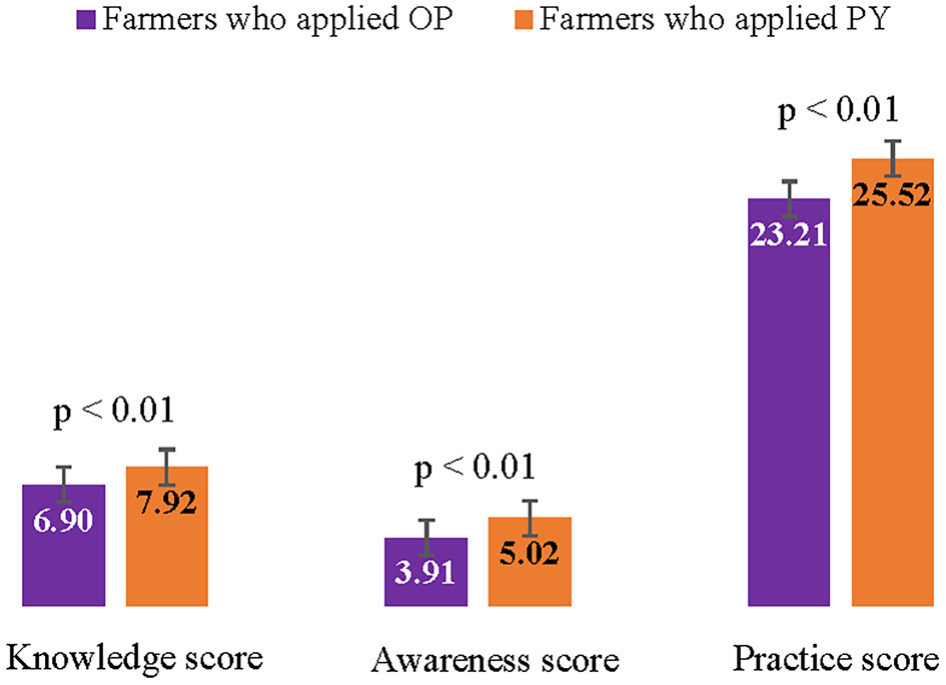
Comparison of knowledge, awareness, and prevention practice scores regarding pesticide use among farmers who applied OP and PY.

Comparisons of the knowledge, awareness, and prevention practice scores regarding pesticide use among farmers who applied OP and PY are presented in Figure 2. The results show that farmers who applied OP had significantly lower knowledge, awareness, and practice scores than those who applied PY. The knowledge scores regarding pesticide use of the total cohort of farmers was 7.3 ± 1.5. For the farmers who applied OP and PY, the knowledge scores regarding pesticide use were 6.9 ± 1.4 and 7.9 ± 1.4, respectively. The awareness scores regarding pesticide use of the total cohort of farmers was 4.4 ± 1.1. For the farmers who applied OP and PY, the awareness scores regarding pesticide use were 3.9 ± 0.9 and 5.2 ± 0.9, respectively. The prevention practice score regarding pesticide use of the total cohort of farmers was 24.2 ± 3.5. For the farmers who applied OP and PY, the prevention practice scores regarding pesticide use were 23.2 ± 3.9 and 25.5 ± 2.5, respectively.

Table 5 presents the prevalence of health symptoms related to pesticide exposure among farmers who applied OP and PY. After adjusting for covariate variables, the farmers who applied OP had a significantly higher prevalence of health symptoms related to pesticide exposure than those who applied PY (OR = 8.29; 95% CI = 1.07–64.45 for chest pain, OR = 6.98; 95% CI = 1.18–40.58 for chest tightness, OR = 27.54; 95% CI = 2.83–268.42 for dry throat, OR = 5.91; 95% CI = 1.62-21.57 for cough, OR = 10.62; 95% CI = 1.88–59.93 for fatigue, OR = 6.76; 95% CI = 1.28–35.75 for paresthesia, OR = 13.84; 95% CI = 1.79–106.76 for poor concentration, OR = 3.75; 95% CI = 1.17–12.05 for short term memory, OR = 8.99; 95% CI = 2.58–31.26 for insomnia, OR = 5.10; 95% CI = 1.15–22.64 for blurry vision, and OR = 5.82; 95% CI = 1.54–21.99 for decreased sex drive). However, the results showed that the farmers who applied PY had a significantly higher prevalence of eye irritation (OR = 0.32; 95% CI = 0.10–0.96) and blisters (OR = 0.13; 95% CI = 0.03–0.62) than those who applied OP.

**Table 5:** Prevalence of health symptoms related to pesticide exposure among farmers who applied OP and PY (*n* = 117).

**Symptoms**	**Total**	**Farmers who applied**	**Farmers who applied**	**Univariate analysis**		**Multivariate analysis^a^**	
	**(*n =* 117)**	**OP (*n =* 67)**	**PY (*n =* 50)**				
				**Crude OR**	**95%CI**	**Adjusted OR**	**95%CI**
**Respiratory**		
Difficulty in breathing	19 (16.2)	7 (10.4)	12 (24.9)	0.37	0.13–1.02	0.60	0.09–4.05
Chest pain	18 (15.4)	15 (22.4)	3 (6.0)	4.52	1.23–16.59^*^	8.29	1.07–64.45^*^
Chest tightness	23 (19.7)	20 (29.9)	3 (6.0)	6.67	1.86–23.96^**^	6.98	1.18–40.58^*^
Heart Palpitations	20 (17.1)	16 (23.9)	4 (8.0)	3.61	1.12–11.58^*^	2.36	0.57–9.84
Dry throat	21 (17.9)	20 (29.9)	1 (2.0)	20.85	2.69–161.62^**^	27.54	2.83–268.42^**^
Cough	43 (36.8)	33 (49.3)	10 (20.0)	3.88	1.67–9.01^**^	5.91	1.62–21.57^**^
**Musculoskeletal**		
Numbness	28 (23.9)	22 (32.8)	6 (12.0)	3.59	1.32–9.69^*^	1.98	0.55–7.14
Cramp	68 (58.1)	42 (62.7)	26 (52.0)	1.55	0.74–3.26	1.97	0.63–6.19
Muscle weakness	26 (22.2)	18 (26.9)	8 (16.0)	1.93	0.76–4.88	4.13	0.97–17.17
**Neurological**
Headache	50 (42.7)	25 (37.3)	25 (50.0)	0.59	0.28–1.25	1.18	0.37–3.71
Dizziness	34 (29.1)	21 (31.3)	13 (26.0)	1.29	0.58–2.94	1.66	0.55–5.03
Vomiting	19 (16.2)	10 (14.9)	9 (18.0)	0.79	0.29–2.14	2.11	0.39–11.40
Fatigue	27 (23.1)	22 (32.8)	5 (10.0)	4.40	1.53–12.64^**^	10.62	1.88–59.93^**^
Eye twitches	43 (36.8)	24 (35.8)	19 (38.0)	0.91	0.43–1.95	1.29	0.41–4.03
Hand tremors	20 (17.1)	15 (22.4)	5 (10.0)	2.59	0.88–7.71	2.59	0.59–11.42
Dysesthesia	7 (6.0)	6 (9.0)	1 (2.0)	4.82	0.56–41.38	1.34	0.04–45.45
Paresthesia	24 (20.5)	18 (26.9)	6 (12.0)	2.69	0.98–7.39	6.76	1.28–35.75^*^
**Epithelial/mucosal surfaces**
Eye irritation	48 (41.0)	23 (34.3)	25 (50.0)	0.52	0.25–1.11	0.32	0.10–0.96^*^
Ulcer/blister	23 (19.7)	9 (13.4)	14 (28.0)	0.39	0.16–1.02	0.13	0.03–0.62^*^
Itchy	9 (7.7)	4 (6.0)	5 (10.0)	0.57	0.14–2.25	0.12	0.006–2.32
Sweating	14 (12.0)	9 (13.4)	5 (10.0)	1.39	0.44–4.46	1.22	0.23- 6.39
**Neurobehavioral**
Poor concentration	20 (17.1)	18 (26.9)	2 (4.0)	8.82	1.94–40.07^**^	13.84	1.79–106.76^*^
Short term memory	76 (65.0)	54 (80.6)	22 (44.0)	5.29	2.32–12.05^**^	3.75	1.17–12.05^*^
Compulsion	44 (37.6)	32 (47.8)	12 (24.0)	2.89	1.29–6.49^*^	1.41	0.46–4.34
Depression	11 (9.4)	6 (5.1)	5 (10.0)	0.89	0.25–3.08	1.81	0.20–1.67
Insomnia	61 (52.1)	48 (71.6)	13 (26.0)	7.19	3.15–16.42^**^	8.99	2.58–31.26^**^
**Other symptoms**
Blurry vision	34 (29.1)	27 (44.3)	7 (14.0)	4.15	1.63–10.57^**^	5.10	1.15–22.64^*^
Diarrhea	15 (12.8)	11 (16.4)	4 (8.0)	2.26	0.67–7.57	6.72	0.77–58.58
Stomach ache	12 (10.3)	9 (13.4)	3 (6.0)	2.43	0.62–9.49	5.48	0.46–65.55
Decreased sex drive	39 (33.3)	31 (46.3)	8 (16.0)	4.52	1.85–11.07^**^	5.82	1.54–21.99^**^

a *adjusted for age, gender, nationality, body mass index, smoking status, alcohol consumption, pesticides group, frequency of pesticide use, years in farm work, perception score, and practice score; OP, organophosphates; PY, pyrethroid**s;** OR, odds ratio; CI, confidence interval;*

* *p < 0.05;*

** *p < 0.01*.

## Discussion

Our study found that the farmers who applied OP had lower knowledge, awareness, and prevention practices regarding pesticide use than those who applied PY. It is possible that gender had an effect on knowledge, awareness, and prevention practice regarding pesticide use. In this study there were more female farmers in the PY grou*p* than in the OP grou*p* (46.0 and 23.9%, respectively). It has been postulated that females take fewer risks and have a higher knowledge and awareness of risk than males (24, 25). Therefore, females are more likely to engage in safety practices than males (25–28). In addition, female farmers are generally more concerned about health effects from pesticide exposure (24, 29). Consistent with previous findings, which showed that females wore long-sleeved shirts or jackets and took a shower immediately after spraying pesticides more than males (25, 30). On the other hand, males do not change out of their work clothes until bedtime and must attend to other farming activities after spraying (25, 30). These findings support the notion that risk knowledge, awareness, and the implementation of self-protective behaviors are linked. In addition, females have a different awareness of risk than males and engage in fewer risky behaviors (27). The first reason could be because of males on the farm played a larger role than females (30). As a result, males find it inconvenient to shower and change clothes immediately after spraying pesticides. The second reason could be due to their hygiene and lifestyle habits (30). Another explanation is that females received more information about pesticide exposure risks than males (25). Females may be more likely to adopt some integrated pest management (IPM) strategies than males and efforts to increase their participation in IPM training include biological control methods, bio-pesticides, organic farming, and other strategies. Female initiatives would be beneficial in terms of reducing pesticide exposure (24, 31, 32). Therefore, these findings suggest that gender could have had an effect on knowledge, awareness, and self-protective behavior in farmers.

Regarding the knowledge of pesticide use, our study found that the farmers had the lowest knowledge with regard to the entry route of pesticides (23.9%), followed by disposal empty of pesticide containers (30.8%) and mixing pesticides to the recommendations given on the label (41.9%). Most farmers perceived that pesticides could enter into the body only through inhalation resulting in a lack of protection from pesticide exposure through other routes (33, 34). Pesticides are commonly accepted to enter the body through skin absorption, ingestion, and inhalation, and farmers' knowledge of this needs to be raised (5, 6). The majority of farmers perceived that empty pesticide containers could be thrown away in agricultural land, trash, and river, and also be cleaned and reused. These findings agreed with several studies about misunderstanding regarding disposal of empty pesticide containers (35–39). These unsafe disposal practices are likely to contaminate the environment and pose a risk to both animal and human health (40). The data also indicated that farmers did not read the recommendations on the pesticide labels before mixing pesticides, and as a consequence pesticide were frequently inappropriately and unnecessarily used (7).

Concerning awareness of pesticide use, our study found that the farmers who had the lowest score of awareness were using PPE while applying pesticides (50.4%), followed by washing spray tanks in a river or waterway (58.1%) and wearing PPE while applying pesticides (63.2%). This finding was consistent with previous studies which found that farmers wore just partial PPE (39, 41). According to the reasons for not wearing protective equipment during pesticide handling, it is uncomfortable, expensive to buy, time-consuming to use, unavailable when needed, and unnecessary in each circumstance (5, 34, 39, 42). This finding agreed with several studies about unawareness of washing spray tanks in a river (35, 43). Most farmers stated they washed application equipment near or into irrigation canals (35). According to the findings of this study, indicating that there is a high risk of environmental contamination during that phase of pesticide handling.

Regarding practice of pesticide use, the lowest scoring practice was changing clothes immediately after pesticide application (6.0%), followed by showering immediately after pesticide application (11.1%) and wearing goggles during pesticide application (14.3%). These findings were consistent with previous studies which found that farmers did not change their clothes until they finished all of their work (44, 45). One possible explanation is that most farmers had a working period of 5–8 h or more than 8 h in their field and nearly half of them had a distance of more than 3 km between farm and residence. Therefore, it is impractical for farmers to shower and change clothes immediately after applying pesticides. With regard to PPE, only 14.3 % of farmers wore goggles, a finding consistent with a systematic review by Sapbamrer and Thammachai (46) which stated that farmers in Asia wore goggles for spraying pesticides 16.1% of the time (46).

A remarkable finding was that the farmers who applied OP had a significantly higher prevalence of health symptoms related to pesticide exposure than those who applied PY. Both OP and PY are toxic to humans (47). Organophosphate inhibits the enzyme acetylcholinesterase (AChE), which breaks down the neurotransmitter acetylcholine (48). Whereas, PY disrupts voltage-sensitive sodium channels to cause malfunctioning of nerve cell membranes (49). However, OP is more toxic to humans than PY due to OP toxicity being irreversible, the phosphorylation of the OP-serin bond, which inhibits AChE is a permanent change. Furthermore, OP is also rapidly absorbed through ingestion, inhalation, and dermal exposure (48). In contrast, PY has a lower rate of dermal absorption and is also rapidly metabolized to non-toxic metabolites (50, 51).

As regards respiratory symptoms, the adjusted prevalence of chest pain, chest tightness, dry throat, and cough in the farmers who applied OP indicated they were significantly higher than those who applied PY (OR = 8.29, 6.98, 27.54, and 5.91, respectively). These findings were consistent with the study by Chakrabory (52) that found respiratory symptoms have been reported in association with OP exposure, including chest tightness (OR = 3.26), sore throat or dry throat (OR = 1.76), and dry cough (OR = 2.83) (52). Furthermore, chest pain, dry throat, and cough have been found to be the most prevalent respiratory symptoms associated with OP exposure (53). OP affect the lungs due to increased acetylcholine, through peripheral muscarinic effects on the airway, nicotinic effects on the respiratory muscles, medulla center effects on the brain, and direct toxic effects on the alveolar-capillary membrane (6, 54). However, pulmonary toxicity of PY has not been studied in humans, despite a recent study suggesting a possible cardiovascular risk (55).

Concerning neurological symptoms, the farmers who applied OP had a significantly higher prevalence of fatigue and paresthesia than those who applied PY (OR = 10.62 and 6.76, respectively). These findings were consistent with the study by Farnham (18) that reported an association between pesticide poisonings and neurological symptoms such as paresthesia or tingling in hands or feet (OR = 3.23), dizziness (OR = 2.38), low energy (OR = 2.33), and tremor of the hands (OR = 3.50) (18). Long-term dermal exposure to OP may cause chronic adverse health effects such as paresthesia and organophosphate-induced delayed polyneuropathy (OPIDP) (56, 57). Symptoms are attributable to sensory effects such as paresthesia or tingling, numbness, and pain and motor affects including fatigue, weakness, and paralysis. It develops 2–3 weeks after the poisoning (57). PY exposure can also cause paresthesia especially following cutaneous exposure or intentional consumption (21, 58, 59). However, human toxicity concerning PY is rarely presented in the literature.

In the case of neurobehavioral symptoms, farmers who applied OP had significantly poorer concentration, short term memory, and insomnia than those who applied PY (OR = 13.84, 3.75, and 8.99, respectively). These findings support previous studies that found that organophosphate poisoning in workers had more psychological and affective problems, difficulty concentrating (OR = 2.07), trouble remembering things (OR = 2.54), and insomnia (OR = 2.53) (18, 60, 61). As a consequence of AChE inhibition, OP exposure causes brain hyperactivity and acetylcholine accumulation (62). These effects are the main causes of neurobehavioral and mood disorders (56, 63).

The result of this study is useful to provide information for health surveillance system and health intervention. However, some limitations should be concerned. Firstly, a cross-sectional study design might not clearly explain some causal relationships. Secondary, recall bias may have been reflected in the information of the farmers' behavior. Thirdly, the questionnaires were based on self-reporting potentially leading to a degree of bias in the data. Fourthly, a small sample size may not be representative of all study populations, resulting in statistical analysis with lower power. Fifthly, a control grou*p* of a worker who was not exposed to pesticides was not recruited to control the association between exposure and effect on the grou*p* of interest. Finally, biomarkers were not used to detect OP and PY exposure or other pesticide exposure. Further research should be conducted in longitudinal studies and biomarker analyses on pesticide health effects.

## Conclusion

This study found that the farmers who applied OP had lower knowledge, awareness, and prevention practices regarding pesticide use than those who applied PY. In addition, farmers who applied OP had a significantly higher prevalence of respiratory, neurological, and neurobehavioral symptoms related to pesticide exposure than those who applied PY. Therefore, it can be concluded that OP had a more harmful impact on human health than PY, resulting in a higher prevalence of pesticide-related illnesses. To address these challenges, long-term training about information of pesticide hazard level should be done to raise awareness and permanently change farmers' behavior. Health professionals should be focused on more effective prevention and intervention programs for individuals, families, and the community. The findings of this study on pesticides' adverse effects are beneficial in motivating farmers to look for other ways to protect their crops. Adoption of biological and cultural practices, reduction of pesticide applications, and the use of reduced-risk pesticides are alternative ways. The policy by government should encourage farmers to use of biological control technology, bio-pesticides, organic farming, and other strategies to ensure long-term sustainability.

## Data Availability Statement

The original contributions presented in the study are included in the article/Supplementary Material, further inquiries can be directed to the corresponding author/s.

## Ethics Statement

The studies involving human participants were reviewed and approved by the Ethics Committee of Chiang Mai University's Faculty of Medicine's Research Ethics in Humans (COM-2563-07707). The patients/participants provided their written informed consent to participate in this study.

## Author Contributions

RS, AJ, JR, ST, and SH: conceptualization. AJ, RS, JR, ST, SH, and KW: methodology. RS and AJ: validation, formal analysis, investigation, data curation, and funding acquisition. RS, ST, SH, JR: resources. AJ: Writing original draft preparation. RS: writing review and editing and project administration. RS, JR, ST, SH: supervision. All authors have read and agreed to the published version of the manuscript.

## Funding

This research and innovation activity is funded by National Research Council of Thailand (NRCT) (Grant No. GSCMU(NRCT)/07/2564) and Faculty of Medicine, Chiang Mai University (Grant No. 059/2564).

## Conflict of Interest

The authors declare that the research was conducted in the absence of any commercial or financial relationships that could be construed as a potential conflict of interest.

## Publisher's Note

All claims expressed in this article are solely those of the authors and do not necessarily represent those of their affiliated organizations, or those of the publisher, the editors and the reviewers. Any product that may be evaluated in this article, or claim that may be made by its manufacturer, is not guaranteed or endorsed by the publisher.
