# Supplementary material for: Differences in Knowledge, Awareness, Practice, and Health Symptoms in Farmers Who Applied Organophosphates and Pyrethroids on Farms
Source: Front Public Health. 2022 Feb 2;10:802810. doi: 10.3389/fpubh.2022.802810 (PMC8847372; doi:10.3389/fpubh.2022.802810)
Supplement: Supplementary file 1 [file Data_Sheet_1.DOCX]

**Appendix 1**. The questionnaire to assess demographic characteristics, agricultural information, knowledge, awareness, practice regarding pesticide use, and health symptoms related to pesticide exposure

**Section1: Demographic characteristics**

1) Gender □ (1) Male □ (2) Female

2) Age …………………years

3) Weight ................ kg Height ...................... cm BMI = .................... kg/m^2^

4) Nationality □ (1) Thai □ (2) Other specify ……………………

5) Education level

□ (1) No education □ (2) Primary education

□ (3) Secondary education □ (4) Bachelor degree

6) Household income (bath/month)

□ (1) < 4,500 □ (2) 4,500-10,000

□ (3) 10,000–15,000 □ (4) > 15,000

7) Smoking status □ (1) Yes □ (2) No

8) Alcohol drinking status □ (1) Yes □ (2) No

9) Health status □ (1) No underlying disease

□ (2) Underlying disease (specify)…………………………….

**Section2: Agricultural information**

10) The number of years of farm work …………………years

11) Type of pesticide

□ (1) Insecticides Common name……………………Trade name……………………

□ (2) Herbicides Common name……………………Trade name……………………

□ (3) Fungicides Common name…………………… Trade name……………………

□ (4) Other specify ……………………………………..

12) Type of sprayer

□ (1) Knapsack sprayer

□ (2) Machine sprayer

13) Frequency of pesticide use

□ (1) 2 times/month or less

□ (2) 1-2 times/week

14) A working hour on farm per day

□ (1) 2-4 hours

□ (2) 5-8 hours

□ (3) > 8 hours

15) Distance between farm and residence

□ (1) < 3 kilometers

□ (2) > 3 kilometers

**Section3: Knowledge and awareness regarding pesticide use**

| **Knowledge regarding pesticide use** | | |
| --- | --- | --- |
| 1) | You should select an appropriate pesticide that is specific to the insects. | ____Yes ____No or I don’t Know |
| 2) | Pesticides can enter into the body through ingestion, inhalation, and dermal contact. | ____Yes ____No or I don’t Know |
| 3) | Pesticides must be mixed according to the label's recommendations. | ____Yes ____No or I don’t Know |
| 4) | Pesticides have an adverse effect on human health. | ____Yes ____No or I don’t Known |
| 5) | Pesticides have an adverse effect on animal health. | ____Yes ____No or I don’t Know |
| 6) | Pesticides have an adverse effect on the environment. | ____Yes ____No or I don’t Know |
| 7) | If pesticides splashed into eyes, you should wash your eyes with water immediately. | ____Yes ____No or I don’t Know |
| 8) | While spraying pesticides, you must wear a mask or a respirator. | ____Yes ____No or I don’t Know |
| 9) | Spraying pesticides at noon is more hazardous to health than spraying pesticides in the morning. | ____Yes ____No or I don’t Know |
| 10) | You should change your clothes after applying pesticides. | ____Yes ____No or I don’t Know |
| 11) | Empty pesticide containers should be disposed of by burying in the ground. | ____Yes ____No or I don’t Know |
| **Awareness regarding pesticide use** | | |
| 12) | Pesticides are unnecessary for increasing productivity. | ____Yes ____No or I don’t Know |
| 13) | You should shower and change your clothes after applying pesticides. | ____Yes ____No or I don’t Know |
| 14) | You should not drink or eat while applying pesticides. | ____Yes ____No or I don’t Know |
| 15) | It is necessary to use personal protection equipment (PPE) while applying pesticides. | ____Yes ____No or I don’t Know |
| 16) | It is easy and practical to wear PPE while applying pesticides. | ____Yes ____No or I don’t Know |
| 17) | The spray tanks should not be washed in a river or waterway. | ____Yes ____No or I don’t Know |

**Section4: Practices regarding pesticide use**

| Before application | | |
| --- | --- | --- |
| 1) | Survey type of pests before buying pesticides | _____Yes _____No |
| 2) | Choose pesticides that are labelled | _____Yes _____No |
| 3) | Read the directions on the pesticide label | _____Yes _____No |
| 4) | Mix pesticides as label prescription | _____Yes _____No |
| 5) | Mix pesticides outdoors | _____Yes _____No |
| 6) | Check spraying equipment | _____Yes _____No |
| 7) | Use gloves when mixing pesticides | _____Yes _____No |
| During application | | |
| 8) | Wear gloves | _____Yes _____No |
| 9) | Wear boots | _____Yes _____No |
| 10) | Wear long-sleeved shirt | _____Yes _____No |
| 11) | Wear long pants | _____Yes _____No |
| 12) | Wear hat | _____Yes _____No |
| 13) | Wear oral or nose mask | _____Yes _____No |
| 14) | Wear goggles | _____Yes _____No |
| 15) | Spray upwind | _____Yes _____No |
| 16) | Do not eat food or drink | _____Yes _____No |
| After application | | |
| 17) | Change clothes immediately | _____Yes _____No |
| 18) | Shower immediately | _____Yes _____No |
| 19) | Wash equipment before storing | _____Yes _____No |

**Section5: Questions regarding health symptoms related to pesticide exposure within 1 month**

| Respiratory symptom | | |
| --- | --- | --- |
| 1) | Difficulty in breathing | _____Yes _____No |
| 2) | Chest pain | _____Yes _____No |
| 3) | Chest tightness | _____Yes _____No |
| 4) | Heart Palpitations | _____Yes _____No |
| 5) | Dry throat | _____Yes _____No |
| 6) | Cough | _____Yes _____No |
| Musculoskeletal symptom | | |
| 7) | Numbness | _____Yes _____No |
| 8) | Cramp | _____Yes _____No |
| 9) | Muscle weakness | _____Yes _____No |
| Neurological symptom | | |
| 10) | Headache | _____Yes _____No |
| 11) | Dizziness | _____Yes _____No |
| 12) | Vomiting | _____Yes _____No |
| 13) | Fatigue | _____Yes _____No |
| 14) | Eye twitches | _____Yes _____No |
| 15) | Hand tremors | _____Yes _____No |
| 16) | Dysesthesia | _____Yes _____No |
| 17) | Paresthesia | _____Yes _____No |
| Epithelial/mucosal surfaces symptom | | |
| 18) | Eye irritation | _____Yes _____No |
| 19) | Ulcer/blister | _____Yes _____No |
| 20) | Itchy | _____Yes _____No |
| 21) | Sweating | _____Yes _____No |
| Neurobehavioral symptom | | |
| 22) | Poor concentration | _____Yes _____No |
| 23) | Short term memory | _____Yes _____No |
| 24) | Compulsion | _____Yes _____No |
| 25) | Depression | _____Yes _____No |
| 26) | Insomnia | _____Yes _____No |
| Other symptoms | | |
| 27) | Blurry vision | _____Yes _____No |
| 28) | Diarrhea | _____Yes _____No |
| 29) | Stomach ache | _____Yes _____No |
| 30) | Decreased sex drive | _____Yes _____No |
